# The culprit of mesalamine intolerance: case series and literature review

**DOI:** 10.1186/s12876-019-1049-2

**Published:** 2019-07-31

**Authors:** Cheng Xie, Runze Quan, Fangjing Hong, Kaifang Zou, Wei Yan, Yu Fu

**Affiliations:** 1 Division of Gastroenterology, Union Hospital, Tongji Medical College, Huazhong University of Science and Technology, Wuhan, 430022 China; 2 Department of Gastroenterology, Huainan Chaoyang Hospital, Huainan, 232007 China; 3 Department of Gastroenterology, Tongji Hospital, Tongji Medical College, Huazhong University of Science and Technology, Wuhan, 430030 China

**Keywords:** Mesalamine intolerance, 5-aminosalicylic acid, Excipients

## Abstract

**Background:**

Mesalamine is a first-line drug in the treatment of inflammatory bowel diseases, while its intolerance occasionally occurs in clinical practice. Most of adverse reactions are due to the active components, which may lead to step-up treatment, but excipients are sometimes regarded as the chief culprit and can be resolved by transferring to other preparations. Thus, distinguishing different kinds of intolerance is extremely important for clinical decision.

**Case presentation:**

Here we reported two cases with mesalamine intolerance. One patient with 5-aminosalicylic acid intolerance had similar adverse reactions to the treatment of different preparations, while another patient with excipients intolerance failed to tolerate Salofalk but could take Pentasa with no symptoms. Meanwhile, clinical manifestations were analysed and the previous reports referring to excipients intolerance were summarized. It is interesting to found that the patients with excipients intolerance mainly presented with acute skin symptoms, such as skin rash, urticaria and angioedema**.** But the adverse effects of 5-ASA in previous reports include fever, headache, rash, nausea, vomiting, dyspepsia, hepatotoxicity, pancreatitis, interstitial nephritis, pneumonitis, pericarditis and so on.

**Conclusions:**

5-aminosalicylic acid and excipients should be taken into consideration together when mesalamine-related adverse events occur. Of note, a diagnosis of excipient intolerance should be paid more attention in the patients with the presentation of acute skin symptoms.

## Background

Mesalamine, one derivative of 5-aminosalicylic acid (5-ASA), has been recommended as the first-line medicine to induce and maintain remission in patients with mild-to-moderately active ulcerative colitis on account of its efficacy and safety [1, 2]. Although mesalamine preparations are extensively well tolerated compared with sulfasalazine for lacking the sulfapyridine moiety, intolerance to mesalamine occasionally occurs, making those patients still confronted with withdrawal of this crucial medicine. However, the causing agent of mesalamine intolerance can be easily misdiagnosed since mesalamine preparations conclude not only 5-ASA, but also inactive components called as excipients. Excipients are inactive ingredients added to pharmaceutical preparations for aiding in the manufacturing process, sustaining the product stability, and giving desired colors and tastes. They are defined by the International Pharmaceutical Excipients Council (IPEC America and IPEC Europe) as “These are the substance(s) other than the API in finished dosage form, which have been appropriately evaluated for safety and are included in a drug delivery system to either aid the processing or to aid manufacture, protect, support, enhance stability, bioavailability or patient acceptability, assist in product identification, or enhance any other attributes of the overall safety and effectiveness of the drug delivery system during storage or use” [3]. Although excipients are considered to be pharmacologically inert, they can still initiate some adverse reactions. To the best of our knowledge, no reports have describing excipients intolerance of mesalamine preparations by now. In this paper, we described two patients with mesalamine intolerance but of 5-ASA and excipients intolerance respectively. Meanwhile, the previous reports referring to excipients intolerance of other drugs were also summarized and analyzed.

## Case presentation

### Case 1

A 50-year-old male patient initially presented with a 1-month history of 3 bouts of mucoid bloody diarrhea per day, lower abdominal pain and crissum discomfort. The ileocolonoscopic findings in the local hospital indicated the colorectal inflammation and bleeding, which was consistent with ulcerative colitis. Then, he was introduced with mesalamine slow release granules (Etiasa) at 4,000 mg/day in our gastroenterology clinic. Unfortunately, he started to develop fever over 40 °C, worse abdominal pain and bloody diarrhea with 10–20 stools movements per day 2 h after mesalamine treatment. He has no history of medications intolerance before Etiasa was considered as the most possible cause and was immediately discontinued at the emergency department. The symptoms subsided after 24 h and disappeared after 72 h.

Subsequently, the patient was admitted to our department for further management. Laboratory examinations revealed increases in C-reactive protein level (CRP) 46.30 mg/L (< 8 mg/L) and erythrocyte sedimentation rate (ESR) 26 mm/h (< 15 mm/h). EB virus DNA (PCR) was positive at 764 copies/mL (< 400 copies/mL) in plasma. Other infectious values were negative, including cytomegalovirus, tuberculin skin tests, T-SPOT.TB test, stool and blood tests for bacterial and fungal pathogens. An ileocolonoscopy was not performed immediately because of sinus bradycardia in the electrocardiogram. Thus, then he carried a Holter monitor for 24 h, and sick sinus syndrome was finally diagnosed. Three days later he was readministered with Etiasa at 500 mg/day with the similar anaphylactic symptoms that promptly disappeared after another suspension of Etiasa. Given an EB virus infection with the patient, oral corticosteroids were not used. Probiotics and glutamine entero-soluble capsule were ultimately prescribed, which attenuated his abdominal pain and bloody diarrhea.

However, the patient self-discontinued those medications and individually took mesalamine enteric-coated tablets (Salofalk) at 500 mg/day, leading to the similar adverse reactions again and readmission to our hospital. Laboratory examinations revealed: CRP 36.70 mg/L and ESR 26 mm/h. Other laboratory indexes were in the normal range. An ileocolonoscopy revealed diffuse hyperemia, swelling and effusion in the colorectal mucosa, with histological features of ulcerative colitis. In view of his intolerance to Etiasa and Salofalk, mesalamine slow release tablets (Pentasa) was subsequently given, which similarly induce the above-mentioned allergic symptoms 2 h later. Considering the hypersensitivity for 5-ASA, he was finally prescribed with rectal corticosteroids to control the symptoms.

### Case 2

A 52-year-old woman initially presented with a 2-month history of 4–5 bouts of bloody diarrhea per day, abdominal discomfort and tenesmus, with deteriorative hematochezia since half a month ago. She was subsequently diagnosed with ulcerative colitis by ileocolonoscopy. Therefore, she was initially prescribed with Salofalk at 2,000 mg/day, which, however, induced generalized maculopapular skin rashes over the trunk and limbs with pruritus.

Then, the patient was admitted to our hospital. Laboratory examinations were: hemoglobin level (Hb) 9.5 g/dl (11.5–15.0 g/dl), CRP 55.10 mg/L, ESR 38 mm/h and serum albumin level 2.59 g/dl (3.5–5.5 g/dl). EB virus and cytomegalovirus tests were negative, as well as stool tests for bacterial and fungal pathogens. An ileocolonoscopy showed moderate to severe inflammation from descending colon to rectum, with histological findings in favor of ulcerative colitis. According to her medical history, the rash was considered to be associated with mesalamine administration, which prompted the withdrawal of Salofalk. Rash and pruritus began to subside within the next 24 h. To further confirm whether the allergic skin reaction was due to Salofalk, the reintroduction was performed, and the similar symptoms emerged unsurprisingly which disappeared after the discontinuation of Salofalk. Thus, mesalamine suppositories (Pentasa) at a dosage of 1000 mg/day was then tried to control the symptoms after seven days, and unexpectedly found with an excellent response. Ten days later, mesalamine slow release tablets (Pentasa) at 1,000 mg/day was added. Subsequently, the dosage of mesalamine tablets increased to 2,000 mg/day for 7 days, next to 3,000 mg/day for one day and eventually maintained at 4,000 mg/day. The patient gradually got better and had no adverse reactions during the administration of Pentasa.

Three months later, an updated ileocolonoscopy demonstrated the remarkable improvement of colonic inflammation. Therefore, mesalamine tablets were reduced to a dose of 3,000 mg/day, still accompanied with mesalamine suppositories at 1,000 mg/day. Then one month after that, mesalamine suppositories were suspended and oral mesalamine was returned to 4,000 mg/day for maintenance treatment. The patient has been well-response with absolute resolution of symptoms.

## Discussion and conclusions

Drug-related adverse reactions are commonly confronted matters in daily medical practice. It is of great significance to distinguish the cause of the drug intolerance since patients were possibly confronted with different therapeutic strategies. As we all know, various mesalamine preparations have been developed and generally applied in daily clinical work, which are better tolerated with fewer adverse effects but still unavoidable. The common adverse effects of mesalamine intolerance include fever, headache, rash with pruritus, nausea, vomiting, and dyspepsia. The rare but severe ones are hepatotoxicity [4, 5], pancreatitis [6, 7], interstitial nephritis [8, 9], pneumonitis [10], and pericarditis [11, 12]. Those symptoms are always dose dependent and can be resolved with decreased dosage [13], while the frequency of these adverse reactions do not increase with increase of mesalamine dosage [14].

It is more likely that intolerance to a kind of medicine always due to the active components, but inactive excipients also shouldn’t be ignored [15–18]. As the cases in our reports, the patient in case 1 with 5-ASA intolerance was confronted with suspension of mesalamine and correspondingly received treatment with rather unsafe steroid, immunosuppressant or costly biologics for induction and maintenance of remission. However, the patient in case 2 just couldn’t tolerate Salofalk but responded well to treatment of Pentasa. To the best of our knowledge, the excipients of Salofalk and Pentasa were not exactly the same, indicating the excipients of Salofalk were more likely to be the cause of the intolerance. Salofalk intolerance does not indicate a global intolerance to all mesalamine preparations. In this case, once 5-ASA was wrongly diagnosed as the chief culprit, the patient would be deprived of this vital drug. Therefore, identification of excipients intolerance may provide better therapeutic alternatives for patients and reduce the frequency of corticosteroids.

However, excipients allergy is often easily to be neglected in clinical work. Though the laboratory procedures for hypersensitivity reaction including skin tests and a rechallenge test are available and effective, it is comparatively complicated since most medications are comprised of various excipients, including colors, flavors, preservative, diluents, and so on. In addition, a rechallenge test sometimes may induce severe adverse effects which could be life-threatening. Under this circumstances, we consider whether it is possible to roughly distinguish the active ingredients and excipients intolerance according to the clinical manifestations of drug intolerance. Thus, a review of literature was conducted. Pubmed and Web of science were searched up to January 2019 using the following MeSH headings and keywords: “drug-related side effects and adverse reactions”, “intolerance”, “adverse effects”, and “excipients”. The language was restricted to English. For Pubmed, all relevant MeSH terms were used. Then, we selected relevant case reports in which the clinical manifestation and definite diagnosis of excipients intolerance were involved. The previous reports for excipients intolerance are summarized in Table 1. It is interestingly found that the drug intolerance from excipients mainly presented with acute skin manifestations, such as skin rash, urticaria and angioedema, while hepatic, renal, pulmonary and gastrointestinal toxicity were not noticed in adverse events of the excipients, which are consistent with our cases. The adverse effects of 5-ASA in previous reports include fever, headache, rash, nausea, vomiting, dyspepsia, hepatotoxicity, pancreatitis, interstitial nephritis, pneumonitis, pericarditis and so on, while the excipients intolerance of mesalamine was not described previously. As far as we know, the presented case is the first describing excipients intolerance of mesalamine preparations in the literature. Thus, when the mesalamine-related adverse reactions occur and manifest as skin symptoms, excipients intolerance should be taken into our first consideration. If the replacement of other mesalamine preparations is effective, it is largely attributable to the excipients intolerance. Nonetheless, in order to confirm the causing agent of mesalamine intolerance, a rechallenge test should be performed during a quiescent phase of the disease.

**Table 1:** A Summary of Studies about Excipients Intolerance

Author (Reference)	medicine name	allergen	clinical manifestation of anaphylaxis
Barni et al. [19]	benzathine benzylpenicillin	soy	an itching papular rash
Barbaud et al. [20]	pills containing paracetamol and a non-steroidal anti-inflammatory drug	carboxymethylcellulose	eczematous rashes
	pills containingpiroxicam and carboxymethylcellulose	carboxymethylcellulose	a maculopapular rash in the trunk
	pills containing levothyroxine and carboxymethylcellulose	carboxymethylcellulose	a chronic generalized urticaria
Bigliardi et al. [21]	Xyloneural and Kenacort® A 40	carboxymethylcellulose	generalized pruritus, urticaria, angioedema in the lips, tachycardia and hypotonia
	carbostesin and Triamcort-Depot®	carboxymethylcellulose	a generalized urticaria, angioedema in the lips, dizziness and breathing problems
	lidocaine and Diprophos®	carboxymethylcellulose	sweating, flush with generalized pruritus, nausea and angioedema in the feet and in the lips
Bircher et al. [22]	lidocaine and Kenacort A10	carboxymethylcellulose	nausea, sweating, thoracic oppression, pruritus, generalized urticaria, and hypotension
Caliskaner et al. [23]	rifampicin	patent blue V (PBV)	macular, itchy rashes in the face, ears, buttocks, elbows and knees
Duenas-Laita et al. [24]	a generic omeprazole capsule	soybean oil	hypotension and difficulty breathing
Field et al. [25]	triamcinolone acetate	carboxymethylcellulose	a rash, urticaria and associated periorbital angioedema
Gooch et al. [26]	warfarin	one of the dyes	recurrent, pruritic maculopapular rashes in the trunk and upper limbs
Koppel et al. [27]	tegretol	FD&C Red 40	rhinorrhea, tearing, and nasal stuffiness
Laing et al. [28]	triamcinolone acetonide	carboxymethylcellulose	lip swelling, wheezing, and an urticarial rash
Millar et al. [29]	erythromycin	the colouring agent E110	some tingling and swelling in the fingers and feet
Moneret-Vautrin et al. [30]	althesin	anti-cremophor EL IgG STS antibodies	cyanosis, an erythema in the whole trunk
Mumoli et al. [31]	furosemide	croscarmellose sodium	an erythematous cutaneous rash with diffuse itching
Rogkakou et al. [32]	ferrous sulphate	orange disperse (Sunset Yellow)	a severe facial erythema with itching and skin oedema
Rubinger et al. [33]	prednisone	acacia	itching with rash and fever
	prednisone	tragacanth	itching with a rash
	prednisone	refused to be tested	itching with arthralgia
Sims-McCallum et al. [34]	6-Mercaptopurine Tablets	cornstarch	a red maculopapular rash in the chest, back, and arms with slightly pruritic
van Hooff et al. [35]	cyclosporin	the solvent	headache, erythema in the head, oedema in the conjunctivae, high blood pressure, and tachycardia

In conclusion, active components are not always responsible for adverse drug reactions, sometimes excipients intolerance need to be noticed, particularly when the adverse reactions are presented as skin manifestations. Otherwise, excipients intolerance may be misdiagnosed as intolerance to the specific active component, which may lead to the change of therapeutic strategies, especially for mesalamine intolerance.

## Data Availability

Not applicable.

## Abbreviations

5-ASA: 5-aminosalicylic acid
CRP: C-reactive protein level
ESR: Erythrocyte sedimentation rate
